# Plant-Based Diets and Ovarian Cancer Risk

**DOI:** 10.3390/nu18030536

**Published:** 2026-02-05

**Authors:** Giovanna Esposito, Federica Turati, Silvia Mignozzi, Fabio Parazzini, Livia S. A. Augustin, Sara Vitale, Jerry Polesel, Luigino Dal Maso, Eva Negri, Carlo La Vecchia

**Affiliations:** 1 Department of Clinical Sciences and Community Health, Dipartimento di Eccellenza 2023–2027, University of Milan, 20133 Milan, Italy; federica.turati@unimi.it (F.T.); silvia.mignozzi@unimi.it (S.M.); fabio.parazzini@unimi.it (F.P.); carlo.lavecchia@unimi.it (C.L.V.); 2 Fondazione IRCCS Ca’ Granda Ospedale Maggiore Policlinico, 20122 Milan, Italy; 3 Epidemiology and Biostatistic Unit, Istituto Nazionale Tumori, IRCCS “Fondazione G. Pascale”, 80131 Naples, Italy; l.augustin@istitutotumori.na.it (L.S.A.A.); sara.vitale@istitutotumori.na.it (S.V.); 4 Cancer Epidemiology Unit, Centro di Riferimento Oncologico di Aviano (CRO), IRCCS, 33081 Aviano, Italy; polesel@cro.it (J.P.); dalmaso@cro.it (L.D.M.); 5 Department of Medical and Surgical Sciences, University of Bologna, 40138 Bologna, Italy; eva.negri@unibo.it

**Keywords:** ovarian cancer, plant-based diet, case-control study, dietary patterns

## Abstract

**Objective**: To assess the relationship between adherence to various plant-based diets, as measured by overall, healthy, and unhealthy plant-based diet indices (PDI, hPDI, uPDI), and ovarian cancer risk. **Methods**: We obtained data on 1031 cases of ovarian cancer and 2411 controls from a case-control study conducted in Italy. PDI, hPDI, and uPDI were calculated using data from a validated food frequency questionnaire. We used logistic regression to calculate the odds ratios (ORs) and their corresponding 95% confidence intervals (CIs) of ovarian cancer for PDI, hPDI, and uPDI, adjusting for several possible confounders. **Results**: PDI and hPDI were inversely related to ovarian cancer risk (OR = 0.70 for the fourth compared to the first quartile, 95% CI: 0.55–0.89, and OR = 0.67, 95% CI: 0.53–0.84, respectively). On the other hand, a higher uPDI was related to a higher risk of ovarian cancer (OR = 1.78, 95% CI: 1.40–2.28). The estimates for a 5-point increment in the indices were 0.88 (95% CI: 0.81–0.95) for PDI, 0.90 (95% CI: 0.83–0.96) for hPDI, and 1.15 (95% CI: 1.07–1.23) for uPDI. Consistent associations for the three indices were observed across strata of age, family history of breast/ovarian cancer, educational level, parity, oral contraceptives use, and menopausal status. **Conclusions**: Plant-based diets favorably influence ovarian cancer risk; plant-based diets characterized by a high intake of unhealthy plant foods are linked to an increased risk. Promoting diets rich in healthy plant foods could support the reduction of ovarian cancer risk.

## 1. Introduction

Ovarian cancer ranks seventh for incidence and sixth for mortality among all malignancies in females [1]. Its incidence has declined over the past two decades, due to the increased use of oral contraceptives (OC), which are well-established protective factors against ovarian cancer [2,3]. However, ovarian cancer remains a major threat to women since there are currently no effective screening tests, and therefore, most patients are diagnosed too late after the disease progressed.

Several risk factors have been identified. Non-modifiable factors include family history, mutations in the BRCA1 and BRCA2 genes, nulliparity, early menarche, and late menopause. Modifiable factors include overweight and obesity, hormone replacement therapy (HRT), and selected lifestyle habits, including smoking and alcohol consumption [3,4,5,6,7,8]. Regarding diet, the latest summary of the association between diet and ovarian cancer risk by the World Cancer Research Fund-American Institute for Cancer Research classified the evidence for several dietary factors as “limited-no conclusion”, for vegetables, fruit, pulses, red and processed meat, carbohydrates, proteins, various types of fats, and vitamins, due to the limited number of studies or the conflicting results across them [9]. Still, the hypothesis that some compounds found in plant foods may reduce the risk has emerged [10,11,12,13].

Phytochemicals, such as flavonoids, polyphenols, lignans, and carotenoids, which are bioactive, non-nutritive compounds found in fruits, vegetables, legumes, and whole grains, have attracted attention due to their potential anti-cancer properties [14,15]. These plant-based compounds modulate the key biological processes involved in carcinogenesis, including antioxidant enzyme activity, reduced oxidative stress, cell cycle arrest, apoptosis, inhibition of gene expression in cancer progression, modulation of signaling pathways, and inhibition of angiogenesis and metastasis [16,17]. These compounds can target multiple molecular and signaling pathways to reverse early metabolic and epigenetic alterations in cancer cells, including the modulation of membrane receptors and enzyme activities such as DNA methyltransferases [18,19]. The potential of chemoprevention through diets rich in plant-based antioxidants is promising, as they can help reduce the risk factors associated with cancer progression [20,21]. Several epidemiological and experimental studies support the protective role of diets rich in these compounds against ovarian cancer [10,11,12].

There has been a growing interest in plant-based diets as a means of improving health and promoting environmental sustainability. A plant-based diet is defined as a dietary pattern that includes a high intake of foods of plant origin, such as fruits, vegetables, legumes, whole grains, nuts, and seeds. Unlike vegetarian or vegan diets, it does not necessarily exclude animal products but encourages the consumption of plant-based foods and the reduction of animal-based foods. Current evidence from observational studies suggests an inverse association between plant-based diets and overall cancer risk [22], particularly of the digestive system [23,24].

Satija et al. [25] derived three indices to measure adherence to plant-based diets. According to all indices, animal-based foods received negative scores. The overall plant-based diet index (PDI) gives positive scores to all types of plant foods, treating them favorably. The healthy plant-based diet index (hPDI) specifically favors the consumption of healthy plant-based foods, such as fruits, vegetables, and whole grains, while penalizing less healthy options, such as refined grains and sweets. Conversely, the unhealthy plant-based diet index (uPDI) favors the consumption of less healthy plant foods and gives negative scores to healthy plant foods.

No studies have comprehensively examined the differential effects of healthy versus less healthy plant-based foods on ovarian cancer. Therefore, important gaps remain in understanding whether overall adherence to plant-based diets or the quality of plant foods may influence ovarian cancer risk. This investigation aims to evaluate the association between overall, healthy, and unhealthy PDIs and the risk of ovarian cancer in a case-control study from Italy.

## 2. Materials and Methods

### 2.1. Study Design and Data Collection

The data used were obtained from a case-control study of ovarian cancer conducted between 1992 and 1999 in multiple regions of Italy, i.e., the greater Milan area and the provinces of Pordenone, Gorizia, and Padua in the North; the Latina province in central Italy; and the Naples metropolitan area in the South [26]. Cases were 1031 females (median age 56, range 18–79 years) with incident, histologically confirmed epithelial ovarian cancer admitted to the major teaching and general hospitals in the study areas. Non-epithelial and borderline ovarian tumors were excluded. Information on stage, as defined by the International Federation of Gynaecology and Obstetrics (FIGO), was available for 587 (57%) cases. The majority (78%) were in an advanced stage (III or IV). Cases were diagnosed with ovarian cancer within one year prior to interview (median time between diagnosis and interview was 1 month). Controls were 2411 females from the same catchment areas and admitted for a wide range of acute, non-neoplastic conditions that were not related to hormonal or gynecological conditions, digestive tract diseases, or long-term dietary changes (e.g., traumas, non-traumatic orthopedic conditions, acute surgical disorders, and other miscellaneous conditions). Females who had undergone bilateral ovariectomy were excluded from the control group. No formal matching was performed. However, controls were selected to be comparable to cases with respect to age (five-year age groups) and study center. Participation rates exceeded 95% for both cases and controls, regardless of catchment area or hospital.

Cases and controls were interviewed face-to-face by centrally trained interviewers using a structured questionnaire. All interviews were conducted in hospital settings. Interviewing nurses were introduced to patients by the attending clinical staff. Proxy interviews were not permitted; all interviews were conducted directly with study participants. The questionnaire included data on socio-demographic and anthropometric characteristics, physical activity, lifetime smoking and drinking habits, personal medical history, family history of cancer, menstrual and reproductive history, and the use of OCs and HRT. The usual diet of the study participants in the two years prior to their cancer diagnosis (for cases) or hospital admission (for controls) was assessed using a reproducible [27] and valid [28] food frequency questionnaire (FFQ). The FFQ covered the average weekly consumption of 78 foods, food groups, and recipes and included questions about intakes of added fats (e.g., olive oil, seed oils, butter, and margarine). Patients were asked to report their average weekly consumption of each item, including occasional consumption (i.e., less than once a week, but at least once a month), which was coded as 0.5 per week. The portion size for 40 food items was defined in ‘natural’ units (e.g., one teaspoon of sugar or one egg), while the portion size for the remaining items was defined as small, average, or large and illustrated with pictures. For fruits and vegetables subject to seasonal variations, consumption during the relevant season and the corresponding duration of consumption were elicited. From FFQ data, the intakes of selected nutrients, food components, and total energy were estimated using an Italian food composition database [29].

### 2.2. Derivation of Plant-Based Diet Indices

We calculated the three indices (i.e., PDI, hPDI, and uPDI) proposed by Satija et al. [25]. In brief, PDIs were derived using 16 food groups: fruits, vegetables, legumes, vegetable oils, tea and coffee, whole grains, and nuts (i.e., the healthy plant foods); refined grains, potatoes, sweets and desserts, and fruit juices/sugar-sweetened beverages (i.e., the less healthy plant foods); and animal fats, dairy products, eggs, fish and seafood, and meat (animal foods).

A score from 1 to 5 was assigned to each food group according to quintiles of consumption (based on servings per day of the control group), with the exception of whole grains, fruit juices/sugar-sweetened beverages, and nuts. For PDI, the highest consumption quintile was assigned a score of 5 for each plant food group (both healthy and less healthy), while the lowest consumption quintile received a score of 1; intermediate consumption quintiles were assigned scores of 4, 3, and 2 (positive scoring). For animal-based food groups, the highest consumption quintile received a score of 1 and the lowest quintile a score of 5; intermediate consumption quintiles were assigned scores of 2, 3, and 4 (reverse scoring). For hPDI, positive scores were given to healthy plant food groups and reverse scores to less healthy plant and animal food groups. The scoring scheme was reversed in the uPDI, with positive scores assigned to less healthy plant food groups and reverse scores to healthy plant and animal food groups.

Classification by quintiles of consumption was not feasible for whole grains, fruit juices/sugar-sweetened beverages, and nuts, since most subjects were non-consumers (i.e., 84.4%, 71.5%, and 99.4%, respectively). For whole grains and sugar-sweetened beverages/fruit juices, patients were classified into three categories: consumers with intake above the median (calculated among controls); consumers with intake below the median; and non-consumers. For whole grains, positive scores of 5, 3, and 1 were assigned in PDI and hPDI, and the reverse scores of 1, 3, and 5 were assigned in uPDI. For fruit juices and sugar-sweetened beverages, positive scores of 5, 3, and 1 were assigned to PDI and uPDI, and the reverse scores of 1, 3, and 5 to hPDI. For nuts, we assigned two points to consumers and one point to non-consumers within PDI and hPDI, and reverse scoring in uPDI.

Compared with the original PDI formulation, the following additional modifications were introduced: (i) we did not consider the “miscellaneous animal-based foods” group, which included a small number of additional animal-derived products and pizza, because most items in this category were not assessed in our FFQ. (ii) Since pizza is traditionally made with yeast-based refined wheat flour dough and a limited number of ingredients, and since animal products account for roughly half or less of its final weight, we allocated half of a pizza portion to the refined grain group and the other half to the dairy group. (iii) Since fruit juices and sugar-sweetened beverages were consumed infrequently by our study population, we combined them into a single food group, whereas in the original formulation, they were treated as separate components.

In addition, as a sensitivity analysis, we derived the hPDI and uPDI by classifying pasta in the healthy plant food group alongside whole grains. In Italy, by law, commercial dry pasta is made with durum wheat semolina (DPR 187/2001), a good source of dietary fiber. It has a low glycemic index and has been associated with several health benefits [30,31].

The FFQ food items included in the PDI components, as well as the derivation method used to calculate the PDIs, are detailed in Supplementary Table S1.

### 2.3. Statistical Analysis

We categorized patients into approximate quartiles of each plant-based diet index using cut-offs derived from controls. Logistic regression models were fitted to calculate the odds ratios (OR) of ovarian cancer and corresponding 95% confidence intervals (CI) for the approximate quartiles of the PDIs (hPDI and uPDI), using the lowest quartile as the reference category. All PDIs were also modeled as continuous variables to estimate the OR for a 5-point increase in the score. The models were adjusted for age, education, center, body mass index (BMI), physical activity, smoking and alcohol habits, total energy intake, family history of ovarian/breast cancer, age at menarche, parity, menopausal status, OC, and HRT use.

We carried out stratified analyses by age, education, parity, menopausal status, OC use, and family history of ovarian/breast cancer. We tested for heterogeneity across strata using the likelihood ratio test, comparing models including and excluding interaction terms between the PDI scores and the stratification variable.

All analyses were performed with SAS version 9.4 and software R version 4.3.1. Statistical significance was set at a *p*-value of less than 0.05 (two-tailed).

## 3. Results

**Table 1** shows the distribution of socio-demographic and other selected characteristics in 1031 ovarian cancer cases and 2411 controls. Compared with controls, cases were more educated and reported more frequently a family history of ovarian and breast cancer.

The OR of ovarian cancer according to the PDI, hPDI, and uPDI are presented in **Table 2**. Significant inverse associations were observed for PDI and hPDI, with ORs for the fourth versus the first quartile of 0.70 (95% CI: 0.55–0.89) and 0.67 (95% CI: 0.53–0.84), respectively. In contrast, a direct association was found for uPDI, with an OR of 1.78 (95% CI: 1.40–2.28) for the fourth versus the first quartile. When the scores were considered as continuous variables, the ORs for a 5-point increment were 0.88 (95% CI: 0.81–0.95) for PDI, 0.90 (95% CI: 0.83–0.96) for hPDI, and 1.15 (95% CI: 1.07–1.23) for uPDI.

Classifying pasta within the healthy plant foods did not materially change the findings on hPDI and uPDI. The hPDI was inversely associated with the risk of ovarian cancer (OR = 0.75 for the fourth *versus* the first quartile; 95% CI: 0.59–0.94; OR = 0.91 for a 5-point increase; 95% CI: 0.85–0.98), and the uPDI was positively associated (OR = 1.60 for the fourth *versus* the first quartile; 95% CI: 1.25–2.05; OR = 1.13 for a 5-point increase; 95% CI: 1.06–1.21).

**Figure 1:**
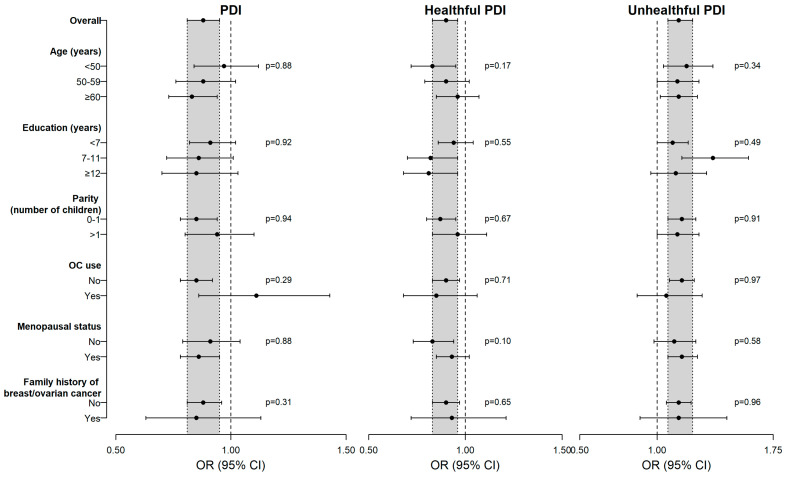
Odds ratios * (OR) and the corresponding 95% confidence intervals (CI) of ovarian cancer for an increment equal to 5 points of plant-based diet indices (PDI), stratified by selected baseline covariates, with *p* values from heterogeneity tests. Italy, 1992–1999. * Estimated from logistic regression models including terms for age, education, center, body mass index, parity, physical activity, smoking, alcohol, total energy intake, family history of breast/ovarian cancer, age at menarche, parity, menopausal status, use of oral contraceptives, and hormone replacement therapy, unless the variable was the stratification factor.

The results of the stratified analyses are presented in **Figure 1**. The associations with the three indices were generally consistent across strata defined by age, education, parity, OC use, menopausal status, and family history of breast/ovarian cancer.

## 4. Discussion

Adherence to the overall and healthful plant-based diets was related to a reduced risk of ovarian cancer, whereas greater adherence to the unhealthful plant-based diet was linked to an increased risk.

An extensive body of the literature has highlighted the potential protective effect of plant-based diets on different cancer types, including breast [32] and digestive tract cancers [33]. As for ovarian cancer, cohort studies observed that adherence to a healthy diet and a higher quality diet before a diagnosis leads to better survival [34,35,36,37]. A meta-analysis of 4 case-control and 12 cohort studies on different healthy dietary scores showed that a high adherence to healthy dietary patterns was linked to reduced ovarian cancer risk and also to better survival outcomes [38].

Although evidence linking plant-based diets to ovarian cancer risk is limited, data are available for the individual components of PDI indices.

Vegetables and fruits, rich in fiber, antioxidants, and phytochemicals, have been studied extensively, but the findings remain inconsistent. While some case-control [26,39,40,41,42] and cohort [43,44,45] studies from Europe, North America, and China reported an inverse association between vegetable intake or consumption of selected vegetables (e.g., green leafy vegetables, tomatoes, garlic, and onions) and ovarian cancer incidence, others [46,47] reported no association. The evidence on the relationship between fruit consumption and ovarian cancer is conflicting. Some studies reported no association [40,43,47], others showed a possible trend towards a positive association [13,26], and other studies found an inverse association [41,42,44].

Dietary fiber is also present in grains and whole grains [30]. In a meta-analysis, total dietary fiber has been related to a lower ovarian cancer risk, with estimates suggesting approximately a 3% risk reduction per 5 g/day increased intake [48]. Although dietary phytochemicals and high-fiber foods have been hypothesized to decrease ovarian cancer risk [10,11,12], findings for specific foods are somewhat inconclusive. A Swedish population-based study reported no significant association for specific phytoestrogen-rich foods such as beans/soy, nuts, berries, and whole-grain bread [49]. In contrast, diets high in refined carbohydrates, characterized by a higher glycemic index, may promote the development of ovarian cancer [50,51].

A favorable effect has been suggested for vegetable oils, including olive oil and specific seed oils such as sunflower, peanut, maize, and soya [52]. Additionally, an elevated intake of total n-6 fatty acids from avocados, vegetables, and nuts was inversely linked to ovarian cancer risk [53]. Legumes have received limited attention. A study suggested that there is an inverse association between consuming legumes and developing ovarian cancer [54]. Similarly, soy foods have been related to a reduced risk of ovarian cancer [55]. Another study investigating the role of diet in female hormone-related cancers also found a possible link with pulses [56], which have been associated with a later menopause onset [57].

As for animal-based foods, there is convincing evidence of an increased risk of ovarian cancer associated with a high intake of total meat, red meat, and processed meat [58]. High consumption of meat, fat, and protein has been linked to a delayed onset of menopause, resulting in prolonged estrogen exposure, which increases the risk of hormone-related cancers, including ovarian cancer [56]. Diets high in red and processed meat have been linked with reduced ovarian cancer survival rates [35,59,60,61]. Dairy products have been hypothesized to increase ovarian cancer risk [62]. However, a pooled analysis of 12 prospective studies showed no association between milk, yoghurt, cheese, ice cream, and total calcium intake and ovarian cancer risk, with only a slight increase in risk for a lactose intake of three or more milk servings/day [63]. Another meta-analysis, which included 18 case-control studies and 11 cohort/nested case-control studies, found that a higher consumption of whole milk was linked to an increased risk of ovarian cancer [64]. The results of epidemiological studies investigating the association between egg consumption and the risk of ovarian cancer remain inconclusive [65,66].

Healthy plant-based diets are characterized by a high consumption of dietary fiber, antioxidants, phytochemicals, and unsaturated fatty acids, which may act synergistically to modulate hormonal regulation, inflammation, oxidative stress, and gut microbiota composition. Dietary fiber promotes short-chain fatty acids production through fermentation by colonic microbiota, influencing the incretin hormones that regulate appetite, insulin sensitivity, and glucose homeostasis, while also reducing systemic pro-inflammatory responses [67,68,69]. In addition, alterations in the human gut microbiome may influence ovarian cancer development by modulating estrogen metabolism. The gut microbiota regulates estrogens by secreting β-glucuronidase, which deconjugates estrogens converting them into their active forms. This consequently affects enterohepatic recycling and systemic estrogen levels, which are critical in hormone-sensitive carcinogenesis [70,71]. Dietary phytochemicals and antioxidants reduce oxidative stress by neutralizing reactive oxygen species and upregulating the endogenous antioxidant defenses, while also modulating inflammatory and cellular stress responses [72,73]. Unsaturated fatty acids, particularly omega-3 polyunsaturated fats, may contribute to reduced systemic inflammation by downregulating pro-inflammatory pathways and improving insulin sensitivity [74]. Lower circulating insulin increases sex hormone-binding globulin synthesis which reduces the bioavailability of free circulating sex hormones. This is another mechanism proposed in relation to low-glycemic-index foods and ovarian cancer risk [75]. Conversely, diets dominated by refined grains, potatoes, sweets, and sugar-sweetened drinks—as reflected by uPDI score—may contribute to metabolic dysfunction, hyperglycemia, hyperinsulinemia, and chronic inflammation, potentially increasing ovarian cancer risk [76,77]. Long-term consumption of high glycemic index carbohydrate foods could lead to chronic hyperinsulinemia, which stimulates insulin-like growth factor 1 (IGF-1) production [78]. A critical role is played by IGF-1 in the stimulation of cell proliferation and the inhibition of apoptosis, processes that can facilitate tumorigenesis and potentially increase the risk of ovarian cancer [79]. Western dietary patterns, based on high consumption of fat, sugar, and ultra-processed foods, may dysregulate gut microbiota composition, further amplifying inflammatory and metabolic disturbances [80].

A strength of the present study is its multicentric design covering different regions of Italy, the high participation, and the use of a reproducible [27] and validated [28] FFQ. Additionally, employing established indices that capture dietary patterns rather than single nutrients or foods provided a comprehensive evaluation of dietary behaviors.

Regarding selection bias, controls hospitalized for chronic, diet-related, hormone-related, or gynecological conditions were excluded. Information bias was limited by conducting interviews for cases and controls in the same hospital setting and using a validated FFQ, and recruiting incident cases likely mitigated this issue. However, we collected information on long-term dietary habits before diagnosis/interview, and not on earlier life diet. Some misclassification of dietary intake due to measurement errors is possible, but it is likely to be non-differential between the cases and controls. Additionally, PDIs were adapted for this study population, for example, by combining fruit juices and sugar-sweetened drinks. Also, the use of scoring systems other than quintiles for some food groups may limit direct comparability with other studies. Finally, we adjust for several correlates of ovarian cancer risk and potential confounding factors.

## 5. Conclusions

Our study indicates that plant-based diets are associated with ovarian cancer risk, with the effect varying according to the quality of the plant foods consumed. Diets rich in healthy plant foods were related to a reduced risk, whereas diets emphasizing unhealthy plant foods were linked to an increased risk. These findings suggest that not all plant-based diets offer the same potential advantages in terms of preventing ovarian cancer. The observed differences according to the quality of the plant food consumed highlight the need to move beyond the simple distinction between plant- and animal-based diets by considering the nutritional quality of plant foods included.

## Figures and Tables

**Table 1:** Distribution of cases with ovarian cancer and controls according to selected characteristics. Italy, 1992–1999.

	Cases N = 1031	Controls N = 2411	*p*-Value
	n (%)	n (%)	
Age (years)			
<50	297 (28.8)	720 (29.9)	
50 to 59	341 (33.1)	694 (28.8)	
≥60	393 (38.1)	997 (41.4)	0.04
Education (years) *			
<7	577 (56.0)	1442 (59.8)	
7 to 11	227 (22.0)	620 (25.7)	
≥12	227 (22.0)	349 (14.5)	<0.01
Parity			
0	184 (17.9)	381 (15.8)	
1	196 (19.0)	473 (19.6)	
≥2	651 (63.1)	1557 (64.6)	0.01
Menopausal status *			
Pre-menopause	346 (33.7)	803 (33.4)	
Post-menopause	683 (66.3)	1603 (66.6)	0.89
Use of oral contraceptives			
No	921 (89.3)	2142 (88.8)	
Yes	110 (10.7)	269 (11.2)	0.68
Family history of breast/ovarian cancer			
No	902 (87.5)	2291 (95.0)	
Yes	129 (12.5)	120 (5.0)	<0.01

* The sum does not add up to the total because of missing values.

**Table 2:** Number of cancer cases, odds ratios * (OR) and the corresponding 95% confidence intervals (CI) of ovarian cancer according to selected plant-based diet indices (PDI). Italy, 1992–1999.

	Ovarian Cancer	
Index (Quartiles)	N (%)	OR (95%CI)
PDI		
I	202 (19.6)	Ref.
II	280 (27.2)	1.02 (0.81–1.29)
III	233 (22.6)	0.86 (0.67–1.10)
IV	316 (30.7)	0.70 (0.55–0.89)
χ^2^ trend (*p*-value)		11.855 (0.01)
5-increment points		0.88 (0.81–0.95)
Healthful PDI		
I	276 (26.8)	Ref.
II	264 (25.6)	0.89 (0.72–1.11)
III	256 (24.8)	0.82 (0.66–1.03)
IV	235 (22.8)	0.67 (0.53–0.84)
χ^2^ trend (*p*-value)		11.715 (0.01)
Unhealthful PDI		
I	193 (18.7)	Ref.
II	251 (24.4)	1.46 (1.15–1.84)
III	296 (28.7)	1.56 (1.24–1.97)
IV	291 (28.2)	1.78 (1.40–2.28)
χ^2^ trend (*p*-value)		20.317 (<0.01)
5-increment points		1.15 (1.07–1.23)

* Estimated from logistic regression models including terms for age, education, center, body mass index, parity, physical activity, smoking, alcohol, total energy intake, family history of breast/ovarian cancer, age at menarche, parity, menopausal status, use of oral contraceptives, and hormone replacement therapy.

## Data Availability

The dataset generated and analyzed during the current study is available from the corresponding author on reasonable request.

## Supplementary Materials

The following supporting information can be downloaded at https://www.mdpi.com/article/10.3390/nu18030536/s1: Table S1: Food items of the food frequency questionnaire (FFQ) constituting the 16 food groups included in the three plant-based diet indices (PDI) and derivation method.

## References

[1] Bray F., Laversanne M., Sung H., Ferlay J., Siegel R.L., Soerjomataram I., Jemal A. (2024). Global cancer statistics 2022: GLOBOCAN estimates of incidence and mortality worldwide for 36 cancers in 185 countries. CA Cancer J. Clin..

[2] Stewart C., Ralyea C., Lockwood S. (2019). Ovarian Cancer: An Integrated Review. Semin. Oncol. Nurs..

[3] Beral V., Doll R., Hermon C., Peto R., Reeves G., Collaborative Group on Epidemiological Studies of Ovarian Cance (2008). Ovarian cancer and oral contraceptives: Collaborative reanalysis of data from 45 epidemiological studies including 23,257 women with ovarian cancer and 87,303 controls. Lancet.

[4] Beral V., Gaitskell K., Hermon C., Moser K., Reeves G., Peto R., Collaborative Group on Epidemiological Studies of Ovarian Cance (2012). Ovarian cancer and smoking: Individual participant meta-analysis including 28,114 women with ovarian cancer from 51 epidemiological studies. Lancet Oncol..

[5] Huang Y.-H., Li J., Luan H., Huang S.-S., Li Y., Li J. (2015). Association between alcohol consumption and the risk of ovarian cancer: A meta-analysis of prospective observational studies. BMC Public Health.

[6] Sung H.K., Ma S.H., Choi J.Y., Hwang Y., Ahn C., Kim B.G., Kim Y.M., Kim J.W., Kang S., Kim J. (2016). The Effect of Breastfeeding Duration and Parity on the Risk of Epithelial Ovarian Cancer: A Systematic Review and Meta-analysis. J. Prev. Med. Public Health.

[7] Whelan E., Kalliala I., Semertzidou A., Raglan O., Bowden S., Kechagias K., Markozannes G., Cividini S., McNeish I., Marchesi J. (2022). Risk Factors for Ovarian Cancer: An Umbrella Review of the Literature. Cancers.

[8] La Vecchia C. (2017). Ovarian cancer: Epidemiology and risk factors. Eur. J. Cancer Prev..

[9] Continuous Update Project Expert Report 2018. Diet, Nutrition, Physical Activity and Ovarian Cancer. https://www.wcrf.org/research-policy/global-cancer-update-programme/.

[10] Khodavandi A., Alizadeh F., Razis A.F.A. (2021). Association between dietary intake and risk of ovarian cancer: A systematic review and meta-analysis. Eur. J. Nutr..

[11] Wang H.F., Yao A.L., Sun Y.Y., Zhang A.H. (2018). Empirically derived dietary patterns and ovarian cancer risk: A meta-analysis. Eur. J. Cancer Prev..

[12] Playdon M.C., Nagle C.M., Ibiebele T.I., Ferrucci L.M., Protani M.M., Carter J., Hyde S.E., Neesham D., Nicklin J.L., Mayne S.T. (2017). Pre-diagnosis diet and survival after a diagnosis of ovarian cancer. Br. J. Cancer.

[13] Crane T.E., Khulpateea B.R., Alberts D.S., Basen-Engquist K., Thomson C.A. (2014). Dietary intake and ovarian cancer risk: A systematic review. Cancer Epidemiol. Biomark. Prev..

[14] Rudzinska A., Juchaniuk P., Oberda J., Wisniewska J., Wojdan W., Szklener K., Mandziuk S. (2023). Phytochemicals in Cancer Treatment and Cancer Prevention-Review on Epidemiological Data and Clinical Trials. Nutrients.

[15] Zhu Y., Sang S. (2017). Phytochemicals in whole grain wheat and their health-promoting effects. Mol. Nutr. Food Res..

[16] Hu R., Kong A.N. (2004). Activation of MAP kinases, apoptosis and nutrigenomics of gene expression elicited by dietary cancer-prevention compounds. Nutrition.

[17] Chen C., Kong A.N. (2005). Dietary cancer-chemopreventive compounds: From signaling and gene expression to pharmacological effects. Trends Pharmacol. Sci..

[18] Pop S., Enciu A.M., Tarcomnicu I., Gille E., Tanas C. (2019). Phytochemicals in cancer prevention: Modulating epigenetic alterations of DNA methylation. Phytochem. Rev..

[19] Pan Y., Mary Peter R., Chou P., Dave P.D., Xu J., Shanner A., Sarwar M.S., Kong A.N. (2025). Cancer-specific Regulation of Metabolic and Epigenetic Pathways by Dietary Phytochemicals. Pharm. Res..

[20] George B.P., Chandran R., Abrahamse H. (2021). Role of Phytochemicals in Cancer Chemoprevention: Insights. Antioxidants.

[21] Wozniak M., Krajewski R., Makuch S., Agrawal S. (2021). Phytochemicals in Gynecological Cancer Prevention. Int. J. Mol. Sci..

[22] DeClercq V., Nearing J.T., Sweeney E. (2022). Plant-Based Diets and Cancer Risk: What is the Evidence?. Curr. Nutr. Rep..

[23] Zhao Y., Zhan J., Wang Y., Wang D. (2022). The Relationship Between Plant-Based Diet and Risk of Digestive System Cancers: A Meta-Analysis Based on 3,059,009 Subjects. Front. Public Health.

[24] Yuan F., Wang L., Nguyen S.M., Shu X.O., Shrubsole M.J., Wen W., Cai Q., Yu D., Zheng W. (2025). Plant-based diets, gut microbiota, blood metabolome, and risk of colorectal, liver and pancreatic cancers: Results from a large prospective cohort study of predominantly low-income Americans. Am. J. Clin. Nutr..

[25] Satija A., Bhupathiraju S.N., Rimm E.B., Spiegelman D., Chiuve S.E., Borgi L., Willett W.C., Manson J.E., Sun Q., Hu F.B. (2016). Plant-Based Dietary Patterns and Incidence of Type 2 Diabetes in US Men and Women: Results from Three Prospective Cohort Studies. PLoS Med..

[26] Bosetti C., Negri E., Franceschi S., Pelucchi C., Talamini R., Montella M., Conti E., La Vecchia C. (2001). Diet and ovarian cancer risk: A case-control study in Italy. Int. J. Cancer.

[27] Franceschi S., Negri E., Salvini S., Decarli A., Ferraroni M., Filiberti R., Giacosa A., Talamini R., Nanni O., Panarello G. (1993). Reproducibility of an Italian food frequency questionnaire for cancer studies: Results for specific food items. Eur. J. Cancer.

[28] Decarli A., Franceschi S., Ferraroni M., Gnagnarella P., Parpinel M.T., La Vecchia C., Negri E., Salvini S., Falcini F., Giacosa A. (1996). Validation of a food-frequency questionnaire to assess dietary intakes in cancer studies in Italy results for specific nutrients. Ann. Epidemiol..

[29] Gnagnarella P., Parpinel M.T., Salvini S., Franceschi S., Palli D., Boyle P. (2004). The update of the Italian food composition database. J. Food Compos. Anal..

[30] Augustin L.S.A., Aas A.M., Astrup A., Atkinson F.S., Baer-Sinnott S., Barclay A.W., Brand-Miller J.C., Brighenti F., Bullo M., Buyken A.E. (2020). Dietary Fibre Consensus from the International Carbohydrate Quality Consortium (ICQC). Nutrients.

[31] Huang M., Li J., Ha M.A., Riccardi G., Liu S. (2017). A systematic review on the relations between pasta consumption and cardio-metabolic risk factors. Nutr. Metab. Cardiovasc. Dis..

[32] Rigi S., Mousavi S.M., Benisi-Kohansal S., Azadbakht L., Esmaillzadeh A. (2021). The association between plant-based dietary patterns and risk of breast cancer: A case-control study. Sci. Rep..

[33] Turati F., Mignozzi S., Esposito G., Bravi F., D’Angelo A., Alicandro G., Garavello W., Augustin L.S.A., Vitale S., Giacosa A. (2025). Indices of healthy and unhealthy plant-based diets and the risk of selected digestive cancers. Clin. Nutr..

[34] Cao F., Wang R., Wang L., Li Y.Z., Wei Y.F., Zheng G., Nan Y.X., Sun M.H., Liu F.H., Xu H.L. (2024). Plant-based diet indices and their interaction with ambient air pollution on the ovarian cancer survival: A prospective cohort study. Ecotoxicol. Environ. Saf..

[35] Wen Z.Y., Liu C., Liu F.H., Wei Y.F., Xu H.L., Wang R., Li X.Y., Li Y.Z., Yan S., Qin X. (2022). Association between pre-diagnostic dietary pattern and survival of ovarian cancer: Evidence from a prospective cohort study. Clin. Nutr..

[36] Zheng G., Gong T.T., Ma Q.P., Wei Y.F., Du Z.D., Zhao J.Q., Zou B.J., Yan S., Liu F.H., Sun M.L. (2023). The association of macronutrient quality and its interactions with energy intake with survival among patients with ovarian cancer: Results from a prospective cohort study. Am. J. Clin. Nutr..

[37] Thomson C.A., Crane T.E., Wertheim B.C., Neuhouser M.L., Li W., Snetselaar L.G., Basen-Engquist K.M., Zhou Y., Irwin M.L. (2014). Diet quality and survival after ovarian cancer: Results from the Women’s Health Initiative. J. Natl. Cancer Inst..

[38] Xu Y., Chen J., Zhao K. (2025). Healthy dietary patterns and ovarian cancer risk and survival: A systematic review and meta-analysis. Front. Nutr..

[39] Edefonti V., Randi G., Decarli A., La Vecchia C., Bosetti C., Franceschi S., Dal Maso L., Ferraroni M. (2009). Clustering dietary habits and the risk of breast and ovarian cancers. Ann. Oncol..

[40] Pan S.Y., Ugnat A.M., Mao Y., Wen S.W., Johnson K.C., Canadian Cancer Registries Epidemiology Research Group (2004). A case-control study of diet and the risk of ovarian cancer. Cancer Epidemiol. Biomark. Prev..

[41] Tang L., Lee A.H., Su D., Binns C.W. (2014). Fruit and vegetable consumption associated with reduced risk of epithelial ovarian cancer in southern Chinese women. Gynecol. Oncol..

[42] Zhang M., Yang Z.Y., Binns C.W., Lee A.H. (2002). Diet and ovarian cancer risk: A case-control study in China. Br. J. Cancer.

[43] Larsson S.C., Holmberg L., Wolk A. (2004). Fruit and vegetable consumption in relation to ovarian cancer incidence: The Swedish Mammography Cohort. Br. J. Cancer.

[44] Kiani F., Knutsen S., Singh P., Ursin G., Fraser G. (2006). Dietary risk factors for ovarian cancer: The Adventist Health Study (United States). Cancer Causes Control.

[45] Schulz M., Lahmann P.H., Boeing H., Hoffmann K., Allen N., Key T.J., Bingham S., Wirfalt E., Berglund G., Lundin E. (2005). Fruit and vegetable consumption and risk of epithelial ovarian cancer: The European Prospective Investigation into Cancer and Nutrition. Cancer Epidemiol. Biomark. Prev..

[46] Bosetti C., Filomeno M., Riso P., Polesel J., Levi F., Talamini R., Montella M., Negri E., Franceschi S., La Vecchia C. (2012). Cruciferous vegetables and cancer risk in a network of case-control studies. Ann. Oncol..

[47] Kolahdooz F., Ibiebele T.I., van der Pols J.C., Webb P.M. (2009). Dietary patterns and ovarian cancer risk. Am. J. Clin. Nutr..

[48] Xu H., Ding Y., Xin X., Wang W., Zhang D. (2018). Dietary fiber intake is associated with a reduced risk of ovarian cancer: A dose-response meta-analysis. Nutr. Res..

[49] Hedelin M., Lof M., Andersson T.M., Adlercreutz H., Weiderpass E. (2011). Dietary phytoestrogens and the risk of ovarian cancer in the women’s lifestyle and health cohort study. Cancer Epidemiol. Biomark. Prev..

[50] Qin B., Moorman P.G., Alberg A.J., Barnholtz-Sloan J.S., Bondy M., Cote M.L., Funkhouser E., Peters E.S., Schwartz A.G., Terry P. (2016). Dietary carbohydrate intake, glycaemic load, glycaemic index and ovarian cancer risk in African-American women. Br. J. Nutr..

[51] Esposito G., Turati F., Parazzini F., Augustin L.S.A., Serraino D., Negri E., La Vecchia C. (2023). Diabetes risk reduction diet and ovarian cancer risk: An Italian case-control study. Cancer Causes Control.

[52] Bosetti C., Negri E., Franceschi S., Talamini R., Montella M., Conti E., Lagiou P., Parazzini F., La Vecchia C. (2002). Olive oil, seed oils and other added fats in relation to ovarian cancer (Italy). Cancer Causes Control.

[53] Ibiebele T.I., Nagle C.M., Bain C.J., Webb P.M. (2012). Intake of omega-3 and omega-6 fatty acids and risk of ovarian cancer. Cancer Causes Control.

[54] Patel L., La Vecchia C., Negri E., Mignozzi S., Augustin L.S.A., Levi F., Serraino D., Giacosa A., Alicandro G. (2024). Legume intake and cancer risk in a network of case-control studies. Eur. J. Clin. Nutr..

[55] Lee A.H., Su D., Pasalich M., Tang L., Binns C.W., Qiu L. (2014). Soy and isoflavone intake associated with reduced risk of ovarian cancer in southern Chinese women. Nutr. Res..

[56] Dunneram Y., Greenwood D.C., Cade J.E. (2019). Diet, menopause and the risk of ovarian, endometrial and breast cancer. Proc. Nutr. Soc..

[57] Dunneram Y., Greenwood D.C., Burley V.J., Cade J.E. (2018). Dietary intake and age at natural menopause: Results from the UK Women’s Cohort Study. J. Epidemiol. Community Health.

[58] Grosso G., La Vignera S., Condorelli R.A., Godos J., Marventano S., Tieri M., Ghelfi F., Titta L., Lafranconi A., Gambera A. (2022). Total, red and processed meat consumption and human health: An umbrella review of observational studies. Int. J. Food Sci. Nutr..

[59] Dolecek T.A., McCarthy B.J., Joslin C.E., Peterson C.E., Kim S., Freels S.A., Davis F.G. (2010). Prediagnosis food patterns are associated with length of survival from epithelial ovarian cancer. J. Am. Diet. Assoc..

[60] Wei Y.F., Sun M.L., Wen Z.Y., Liu F.H., Liu Y.S., Yan S., Qin X., Gao S., Li X.Q., Zhao Y.H. (2022). Pre-diagnosis meat intake and cooking method and ovarian cancer survival: Results from the Ovarian Cancer Follow-Up Study (OOPS). Food Funct..

[61] Nagle C.M., Dixon S.C., Jensen A., Kjaer S.K., Modugno F., deFazio A., Fereday S., Hung J., Johnatty S.E., Australian Ovarian Cancer Study G. (2015). Obesity and survival among women with ovarian cancer: Results from the Ovarian Cancer Association Consortium. Br. J. Cancer.

[62] Cramer D.W., Harlow B.L., Willett W.C., Welch W.R., Bell D.A., Scully R.E., Ng W.G., Knapp R.C. (1989). Galactose consumption and metabolism in relation to the risk of ovarian cancer. Lancet.

[63] Genkinger J.M., Hunter D.J., Spiegelman D., Anderson K.E., Arslan A., Beeson W.L., Buring J.E., Fraser G.E., Freudenheim J.L., Goldbohm R.A. (2006). Dairy products and ovarian cancer: A pooled analysis of 12 cohort studies. Cancer Epidemiol. Biomark. Prev..

[64] Liao M.Q., Gao X.P., Yu X.X., Zeng Y.F., Li S.N., Naicker N., Joseph T., Cao W.T., Liu Y.H., Zhu S. (2020). Effects of dairy products, calcium and vitamin D on ovarian cancer risk: A meta-analysis of twenty-nine epidemiological studies. Br. J. Nutr..

[65] Zeng S.T., Guo L., Liu S.K., Wang D.H., Xi J., Huang P., Liu D.T., Gao J.F., Feng J., Zhang L. (2015). Egg consumption is associated with increased risk of ovarian cancer: Evidence from a meta-analysis of observational studies. Clin. Nutr..

[66] Keum N., Lee D.H., Marchand N., Oh H., Liu H., Aune D., Greenwood D.C., Giovannucci E.L. (2015). Egg intake and cancers of the breast, ovary and prostate: A dose-response meta-analysis of prospective observational studies. Br. J. Nutr..

[67] McLoughlin R.F., Berthon B.S., Jensen M.E., Baines K.J., Wood L.G. (2017). Short-chain fatty acids, prebiotics, synbiotics, and systemic inflammation: A systematic review and meta-analysis. Am. J. Clin. Nutr..

[68] Fu J., Zheng Y., Gao Y., Xu W. (2022). Dietary Fiber Intake and Gut Microbiota in Human Health. Microorganisms.

[69] Portincasa P., Bonfrate L., Vacca M., De Angelis M., Farella I., Lanza E., Khalil M., Wang D.Q., Sperandio M., Di Ciaula A. (2022). Gut Microbiota and Short Chain Fatty Acids: Implications in Glucose Homeostasis. Int. J. Mol. Sci..

[70] Tahri A., Amedei A. (2025). Unraveling the links between estrogen and gut microbiota in sex-hormone driven cancers. World J. Clin. Oncol..

[71] Baker J.M., Al-Nakkash L., Herbst-Kralovetz M.M. (2017). Estrogen-gut microbiome axis: Physiological and clinical implications. Maturitas.

[72] Wu S., Liao X., Zhu Z., Huang R., Chen M., Huang A., Zhang J., Wu Q., Wang J., Ding Y. (2022). Antioxidant and anti-inflammation effects of dietary phytochemicals: The Nrf2/NF-kappaB signalling pathway and upstream factors of Nrf2. Phytochemistry.

[73] Hegazy A.M., El-Sayed E.M., Ibrahim K.S., Abdel-Azeem A.S. (2019). Dietary antioxidant for disease prevention corroborated by the Nrf2 pathway. J. Complement. Integr. Med..

[74] Khalili L., Valdes-Ramos R., Harbige L.S. (2021). Effect of n-3 (Omega-3) Polyunsaturated Fatty Acid Supplementation on Metabolic and Inflammatory Biomarkers and Body Weight in Patients with Type 2 Diabetes Mellitus: A Systematic Review and Meta-Analysis of RCTs. Metabolites.

[75] Augustin L.S., Franceschi S., Jenkins D.J., Kendall C.W., La Vecchia C. (2002). Glycemic index in chronic disease: A review. Eur. J. Clin. Nutr..

[76] Augustin L.S.A., Kendall C.W.C., Jenkins D.J.A., Willett W.C., Astrup A., Barclay A.W., Bjorck I., Brand-Miller J.C., Brighenti F., Buyken A.E. (2015). Glycemic index, glycemic load and glycemic response: An International Scientific Consensus Summit from the International Carbohydrate Quality Consortium (ICQC). Nutr. Metab. Cardiovasc. Dis..

[77] Vitale S., Palumbo E., D’Angelo A., Di Maso M., Polesel J., Grimaldi M., Porciello G., Luongo A., Pica R., Crispo A. (2025). Healthful and Unhealthful Plant-Based Diets and Their Association with Cardiometabolic Targets in Women Diagnosed with Breast Cancer: A Cross-Sectional Analysis of a Lifestyle Trial. Nutrients.

[78] Cohen D.H., LeRoith D. (2012). Obesity, type 2 diabetes, and cancer: The insulin and IGF connection. Endocr. Relat. Cancer.

[79] Yu H., Rohan T. (2000). Role of the insulin-like growth factor family in cancer development and progression. J. Natl. Cancer Inst..

[80] Rondinella D., Margarita E., Raol P.C., Galli F.S., Severino A., Porcari S., Mele M.C., Gasbarrini A., Cammarota G., Rinninella E. (2026). The impact of diet on gut microbiome composition: Implications for immune-mediated diseases. Clin. Immunol. Commun..

