# Supplementary material for: Plant-Based Diets and Ovarian Cancer Risk
Source: Nutrients. 2026 Feb 5;18(3):536. doi: 10.3390/nu18030536 (PMC12899281; doi:10.3390/nu18030536)
Supplement: Supplementary file 1 [file nutrients-18-00536-s001.zip › nutrients-4091569-supplementary.pdf]

Table S1. Food items of the food frequency questionnaire (FFQ) constituting the 16 food groups included in the three plant-based diet indices (PDI) and derivation method.

	FFQ food items	Derivation method		
		PDI	Healthful PDI	Unhealthful PDI
**Plant food groups**				
Healthy				
Whole grains[a]	Whole wheat bread	1: non-consumers, 3: <median, 5: ≥median	1: non-consumers, 3: <median, 5: ≥median	5: non-consumers, 3: <median, 1: ≥median
Fruit	Apples and pears; bananas; kiwi; citrus fruits; peaches, apricots and plums; melon; grapes; strawberries and cherries; unsweetened fruit juices; 1/2 cooked fruit	1 to 5	1 to 5	5 to 1
Raw and cooked vegetables	Green and red salad; raw carrots; cooked carrots; onions; artichokes; cruciferae; spinach/other greens; tomatoes; salad with carrots, cucumbers, peppers; zucchini, eggplants and peppers (cooked); 1/4 vegetable soup or legume soup with pasta	1 to 5	1 to 5	5 to 1
Nuts	Open question	1: non-consumers, 2: consumers	1: non-consumers, 2: consumers	2: non-consumers, 1: consumers
Legumes	Peas, beams, chickpeas, lentils; 1/4 vegetable soup or legume soup with pasta	1 to 5	1 to 5	5 to 1
Vegetable oils	Grams of olive oil, sunflower oil, peanut oils and other vegetable oils (derived from recipes of the FFQ and based on the fat intake patterns declared by the subjects)	1 to 5	1 to 5	5 to 1
Tea & Coffee	Tea, coffee, decaffeinated coffee, cappuccino	1 to 5	1 to 5	5 to 1
Unhealthy				
Refined grains[a]	Bread; crackers, bread sticks, melba toast; maize; risotto; pasta/rice seasoned with butter or oil; pasta/rice seasoned with tomatoes sauce; 1/2 pasta/rice with ragù sauce; pasta/rice with pesto; 1/2 lasagna, cannelloni, tortellini with meat filling; light soup with pasta; 1/2 vegetable soup or legume soup with pasta; 1/2 pizza	1 to 5	5 to 1	1 to 5
Potatoes	Boiled potatoes; fried or roasted potatoes	1 to 5	5 to 1	1 to 5
Sugar sweetened beverages and fruit juices	Sweetened fruit juices; soft drinks	1: non-consumers, 3: <median, 5: ≥median	5: non-consumers, 3: <median, 1: ≥median	1: non-consumers, 3: <median, 5: ≥median
Sweets and desserts	Biscuits; croissants and doughnuts; pastry, doughnuts with cream or custard; cakes; fruit or jam pies, fruit tarts; chocolate candies; candies; honey and jam	1 to 5	5 to 1	1 to 5
**Animal food groups**				
Animal fats	Grams of butter (derived from recipes of the FFQ and based on the fat intake patterns declared by the subjects)	5 to 1	5 to 1	5 to 1
Dairy	Whole milk; partially skimmed milk; skimmed milk; yogurt; 1/2 cappuccino; ricotta and mozzarella cheese; other cheeses; 1/3 any type of cheese in addition or snack; 1/15 grated cheese (1 teaspoon); ice-cream; 1/2 pizza	5 to 1	5 to 1	5 to 1
Eggs	Boiled/poached and raw eggs; fried eggs and omelettes	5 to 1	5 to 1	5 to 1

Fish or Seafood	Boiled or broiled fish/molluscs; fried fish/molluscs; canned tuna and sardines	5 to 1	5 to 1	5 to 1
Meat	Boiled or broiled chicken/turkey; roasted, fried or stewed chicken/turkey and rabbit; steak/roast-beef/lean ground beef, veal and horse meat; boiled beef; beef and veal stew/meatballs; Wiener Schnitzel; pork chop/paillard or pork roast; liver; prosciutto/lean processed meat; ham; salami/sausages, bacon and hot dog; 1/2 pasta/rice with ragù sauce; 1/2 lasagna, cannelloni, tortellini with meat filling	5 to 1	5 to 1	5 to 1

[a]Pasta added together with whole grains in a sensitivity analysis.
